# Thermal conductivity of cryoprotective agents loaded with nanoparticles, with application to recovery of preserved tissues and organs from cryogenic storage

**DOI:** 10.1371/journal.pone.0238941

**Published:** 2020-09-17

**Authors:** Lili E. Ehrlich, Zhe Gao, John C. Bischof, Yoed Rabin

**Affiliations:** 1 Department of Mechanical Engineering, Carnegie Mellon University, Pittsburgh, Pennsylvania, United States of America; 2 Department of Mechanical Engineering, University of Minnesota, Minneapolis, Minnesota, United States of America; 3 Department of Chemistry, University of Minnesota, Minneapolis, Minnesota, United States of America; Oak Ridge National Laboratory, UNITED STATES

## Abstract

The objective of this study is to provide thermal conductivity data for CPA-based nanofluids for the benefit of the analyses of cryopreservation by vitrification. Thermal conductivity measurements were conducted using a hot-wire technique on an experimentation platform of the cryomacroscope, to correlate measurements with observed physical effects such as crystallization and fracturing. Tested materials in this study include the CPA cocktails M22, VS55, DP6, and DP6+sucrose. Nanofluids in this study include the above CPA cocktails as base solutions, when mixed with either iron-oxide nanoparticles (IONP) or silica-coated iron-oxide nanoparticles (sIONP). Results of this study demonstrated the addition of sIONP to any of the CPA cocktails tested did not significantly affect its thermal conductivity, its tendency to vitrify or, conversely, its tendency to form rewarming phase crystallization (RPC). Fractures were observed with cryomacroscopy at the onset of rewarming for DP6+sIONP under carefully controlled rewarming conditions without RF activation, despite the inherent opacity of the sIONP solutions. It is likely that using RF heating in order to accelerate rewarming while unifying the temperature distribution would prevent fracture and RPC. However, sIONP were not activated in this study, as the RF heating mechanism would interfere with thermal conductivity measurements. The addition of IONP to DP6 appears to hinder the tendency of the CPA to vitrify, which is a detrimental effect. But it is unlikely that uncoated nanoparticle solutions will be used in practical applications.

## Introduction

The ability to replace organs and tissues on demand could save or improve millions of lives each year globally and create public health benefits on par with curing cancer [1]. A key factor that limits the availability of recovered organs from donors is their limited shelf life, which is derived from current storage methods and practices. Cryopreservation–the preservation of tissues and organs in very low temperatures–presents the only promising alternative for long-term storage [2], where mass transport is arrested, effectively halting the natural degradation of the biological material at any relevant timescale. This suspended state could facilitate organ transportation, improve donor-recipient matching, prepare the immune system of the recipient to decrease rejection, and even create an opportunity for organ repair. In turn, these expanded possibilities can improve the quality and the likelihood for success of transplantation, while decreasing the associated risks and costs.

Classical methods of cryopreservation essentially revolve around loading the tissue with cryoprotective agents (CPAs) to control ice formation during cooling. These methods have demonstrated success in small and less complex biological systems (μm to mm) such as sperm [3] or cornea [4], or when the mechanical function has a higher priority than viability as is the case with heart valves [5]. In order to avoid ice formation—the cornerstone of cryoinjury [6], cryopreservation of larger specimens and organs requires alternative approaches, with vitrification (*vitreous* in Latin means *glass*) as the most promising. This approach relies on the observation that the viscosity of the CPA increases with the concentration and the decreasing temperature. When the specimen is loaded with a higher concentration CPA and cooled fast enough, such that the time scale for crystallization is longer than the cooling period, the specimen is trapped in an amorphous (glassy) state. While vitrification is a promising approach for larger size cryopreservation, it has its own unique challenges [7].

The current study focuses on a recent technological development for volumetric heating of the specimen, to addresses three of the unique challenges associated with recovery of vitrified material from cryogenic storage: (i) decreasing the likelihood for crystallization during rewarming, (ii) reducing the toxicity potential of the CPA, and (iii) alleviating the conditions leading to structural damage due thermomechanical stress [8]. Here, volumetric heating is achieved by mixing iron oxide nanoparticles (IONP) with the CPA solution and placing the specimen in a radiofrequency (RF) magnetic field [9], where the process is referred to as *nanowarming*.

While physical demonstration of nanowarming can be achieved with IONP [10], IONP will eventually aggregate and fall out of the CPA solution. This necessitated the coating for IONP with mesoporous silica (msIONP) [11], which was then used successfully for nanowarming of cells and simples tissues up to 50 mL [9]. Unfortunately, producing msIONP is laborious and costly in gram quantities, which would be needed for nanowarming scale-up to larger tissues and organs. Thus, a simpler and cheaper sol-gel coating with micro-porous silica (sIONP) has been developed, which is operationally equivalent to msIONP for nanowarming These sIONP can be scaled up to > 1400 mg Fe/batch for nanowarming of large tissues and organs [12].

Specifically, this study focuses on thermal conductivity measurements of a battery of CPAs mixed with uncoated (IONP, EMG-308 Ferrotec) and coated nanoparticles (sIONP, silica-coated EMG-308 Ferrotec). The obtained data is essential for computer simulations that will be used to gain insight into the cryopreservation process and to optimize cryopreservation protocols. These simulations may address thermal effects, kinetics of crystallization, and thermomechanical stress development. Furthermore, the sensitive thermal conductivity measurement system used in the current study provides means to investigate the effect of the presence of nanoparticles on ice nucleation in the specific practical vitrification conditions.

## Materials and methods

### Experimental setup

The experimental setup used in this study has been presented previously [13, 14] and is briefly described here for the completeness of presentation. The setup consists of a visualization device, termed the *scanning cryomacroscope* [15], integrated with a thermal conductivity measurement apparatus, based on a transient hot-wire method [16, 17]. The scanning cryomacroscope is used to ensure vitrification and to facilitate observations of adverse physical events in real time, such as ice formation, thermomechanical stress, and fracturing in the CPA. The transient hot wire setup is designed to make measurements simultaneously such that they may be paired to physical events captured by the cryomacroscope.

**Fig 1:**
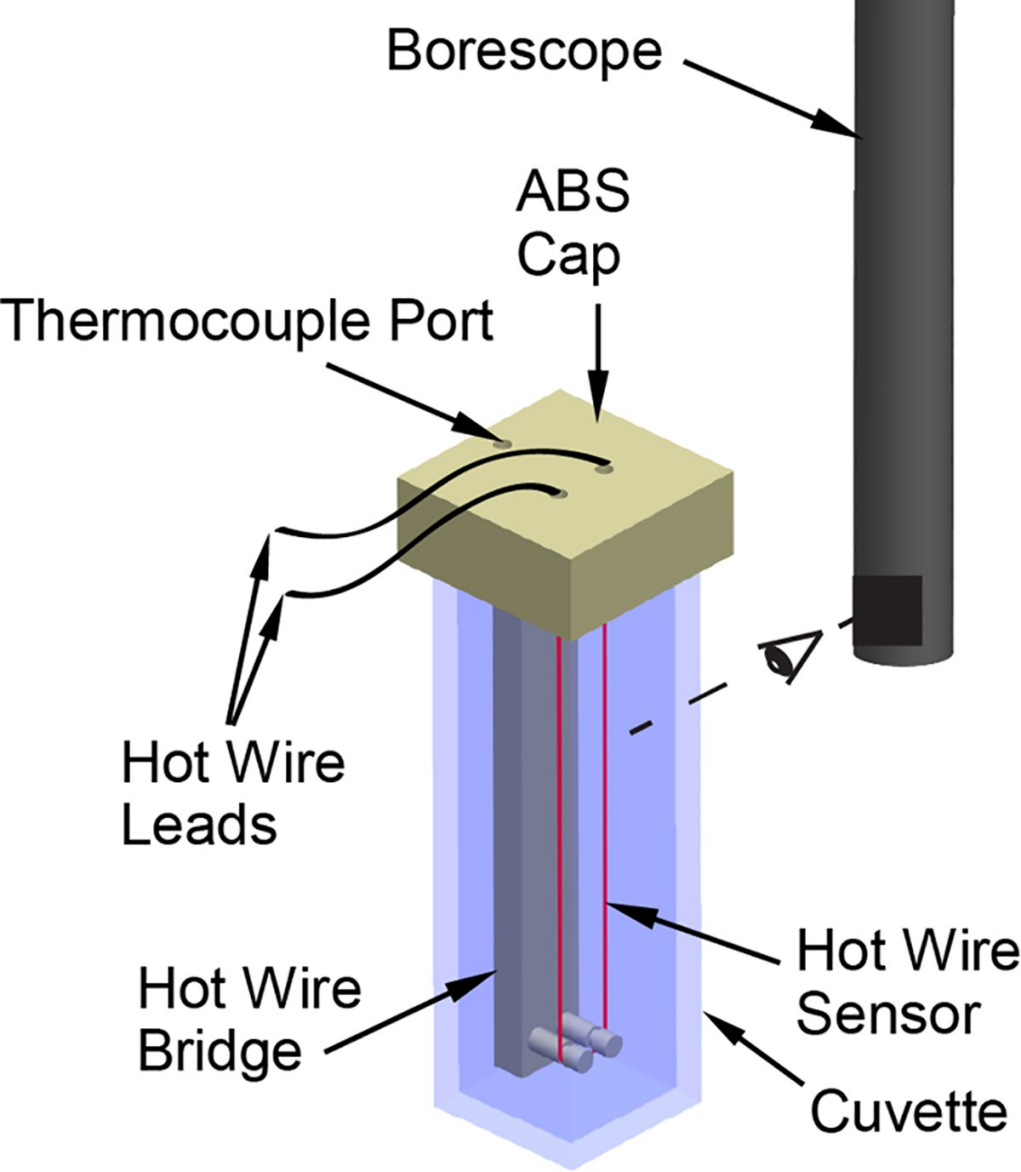
Schematic illustration of the transient hot-wire sensor setup for thermal conductivity measurements; the dashed line represents the direction of visualization by the scanning cryomacroscope [6].

Fig 1 displays a schematic illustration of the transient hot-wire sensor immersed in the CPA solution sample. The sensor and solution are contained in a cuvette (a rectangular vial with superior optical properties), which is positioned on the stage of the scanning cryomacroscope. The cryomacroscope is designed to replace the lid of a commercially available controlled-rate cooling chamber (Kryo 10–20 controller, Planar Ltd., UK) during experiments, while the experimental stage of the cryomacroscope, including the cuvette, are positioned within the cooling chamber (Kryo 10–16 chamber, Planar Ltd., UK).

A U-shaped platinum wire, having a length of 75 mm, serves as the sensor and is held in place by an 3D-printed holder and cuvette cap in one unit (made of ABS). During measurements, the wire acts as both a heater and a resistance temperature sensor. A constant current source (Model 6221, Keithley Instruments, Inc., Ohio) activates a Heaviside step function Joule heating in the wire. As the wire heats up, the resistance change is measured with a digital multimeter (Model 34401A, Keysight Technologies, Inc., Santa Rosa, CA). This resistance change, Δ*R*, is correlated with the wire temperature change, Δ*T*:

$$\Delta T=\frac{\Delta R}{\beta R_{ref}}$$ (1)

where *β* is the coefficient of thermoresistance of platinum corresponding to a reference resistance specific to an individual sensor, *R*_ref_.

The thermal conductivity of the surrounding material is extracted from the temperature response of the wire. Here, a curve is fitted to the temperature response data in the form of the analytical solution to the heat diffusion problem, yielding the measured thermal conductivity:

$$k_{sample}=\frac{q/4\pi }{d(\Delta T)/d(\ln t)}$$ (2)

where *q* is the heat generation rate per unit length in the wire and *t* is total time from electrical current activation. Further details regarding this technique are presented in [13, 18]. Based on the thermal analysis presented in [18], the standard deviation of the temperature variation along the hotwire sensor during rewarming is less than 5% of the its average temperature, which supports the underlying approximation of uniform temperature distribution when using Eqs (1) and (2).

### Thermal protocol

**Fig 2:**
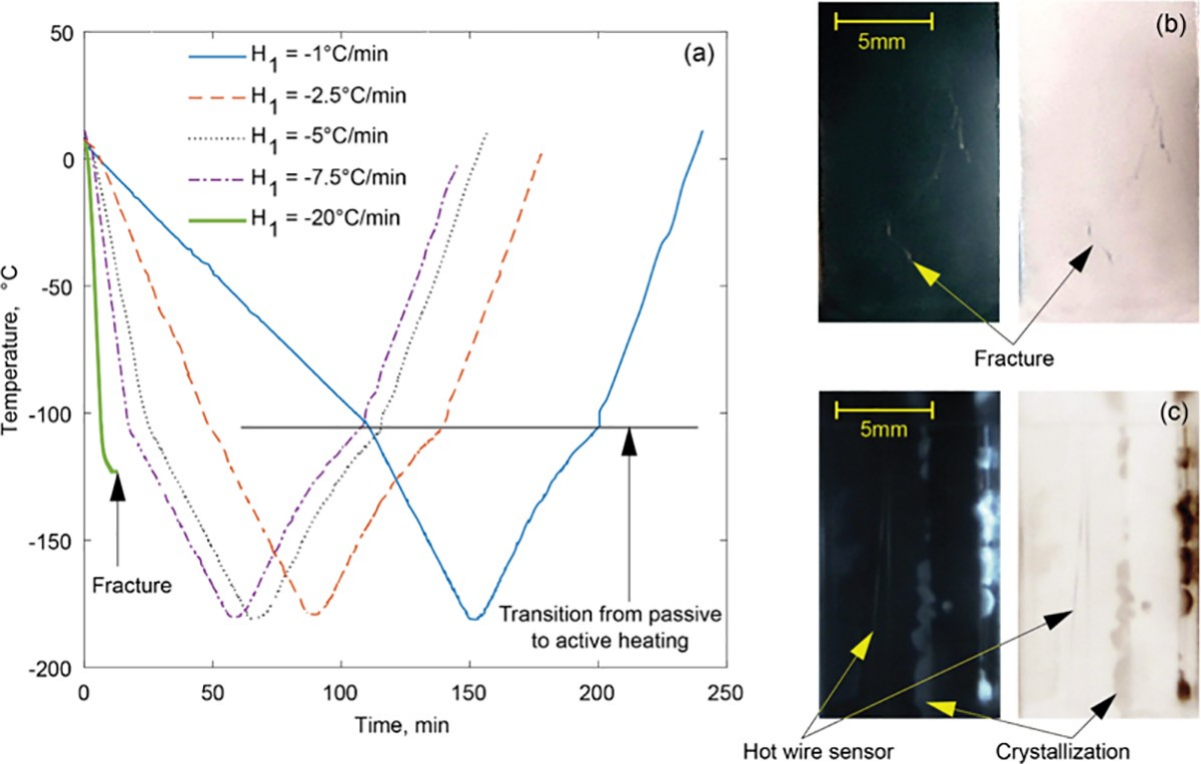
(a) Representative thermal histories, where *H*_1_ refers to the initial cooling rate between 12ºC and -100ºC, including experiments on DP6 + IONP (*H*_1_ = -1 to -7.5°C/min) and DP6+sIONP (*H*_1_ = -20°C/min), and where the reported temperature was measured at the inner surface of the cuvette. (b) A cryomacrograph of fractured DP6+sIONP (occurring at -123°C with *H*_1_ = -20°C/min). (c) A cryomacrograph of partially crystallized VS55 in the absence of nanoparticles. Both cryomacrographs are presented in regular color scheme and a negative color scheme, which may enhance physical events.

The thermal protocol applied in this study is consistent with previous studies [13–15, 19–21], comprising of six steps with reference to Fig 2: (i) precooling the system to 12°C and purging the chamber from moisture and to avoid condensation on the cryomacroscope optics; (ii) cooling at a variable rate of *H*_1_ between -1°C/min and -20°C/min down to a temperature of -100°C (roughly 20°C above the glass transition temperature for the specific tested solutions); (iii) cooling at a rate *H*_2_ = -2°C/min down to a storage temperature of *T*_s_ = -180°C; (iv) holding the system for a time *t*_s_ until it approaches thermal equilibrium; (v) passive rewarming at a rate of *H*_3_ up to -100°C (averaging 1.7°C/min); and (vi) controlled-rate rewarming at a rate of *H*_4_ = 3°C/min.

Measurements were taken during the rewarming portion of the cryoprotocol, where temperature control is of higher quality and uncertainty in measurements is lower. Measurements were taken every 40 s, where each temperature curve fit corresponds to 0.5 s of Joule heating and 30 data points [13]. Temperature changes in the bulk sample due to the rewarming process were subtracted from the temperature response data of the transient hot wire. The uncertainty resulting from this method is ±0.03 W/m-K [13].

Although nanoparticles are present in the solution, RF rewarming was not applied during experimentation as the current study is focused on the physical properties of the mixture.

### Materials tested

This study is focused on the CPA cocktails VS55, DP6, and M22. These CPA solutions have been subjects of previous investigations [9, 14, 20–27] and are now studied with the addition of iron oxide nanoparticles (IONP) and silica-coated IONP (sIONP). Thermal conductivity measurements of pure VS55 and pure M22 are presented here for the first time. Also measured for the first time is DP6 with the addition of 0.5 M sucrose (17.1% by volume), which is a solution of recent interest and investigation [28].

VS55 is a cocktail of 242.14 g/ℓ dimethyl sulfoxide (DMSO) (3.1 M), 168.38 g/ℓ propylene glycol (2.2 M), 139.56 g/ℓ formamide (3.1 M), 2.4 g/ℓ hepes, in EuroCollins solution. The EuroCollins is essentially a vehicle solution including: 34.95 g/L dextrose, 7.3 g/L K_2_HPO_4_, 2.04 g/L KH_2_PO_4_, 1.12 g/L KCl, 0.84 g/L NaHCO_3_. DP6 is similar to VS55 excepting formamide, comprising: 234.4 g/ℓ DMSO (3 M), 228.3 g/ℓ propylene glycol (3 M), 2.4 g/ℓ hepes, in EuroCollins solution. M22 is a commercially available CPA solution from 21^st^ Century Medicine and contains a total of 9.345 M of cryoprotectants, comprising on a weight basis: 22.3% DMSO, 12.86% formamide, 16.84% ethylene glycol, 3% N-methylformamide, 4% 3-methoxy-1,2-propanediol, 2.8% polyvinyl pyrrolidone K12, 1% X-1000, and 2% Z-1000, where the latter two compounds are known as synthetic ice blockers (SIBs) [24].

DP6, VS55 and M22 were mixed with 11 mg Fe/mℓ solution (0.42% Fe_3_O_4_ by volume), amounting to a total of 220 mg sIONP/mℓ solution. DP6 and VS55 were also mixed with 18 mg Fe/mℓ solution and 14 mg Fe/mℓ solution, respectively (0.70% and 0.54% Fe_3_O_4_ by volume). All nanoparticle solutions were sonicated with a probe-type sonicator for a period of 5 minutes total (4 second intervals with 2 second pauses) before each experimental session.

### Nanoparticle synthesis

**Fig 3:**
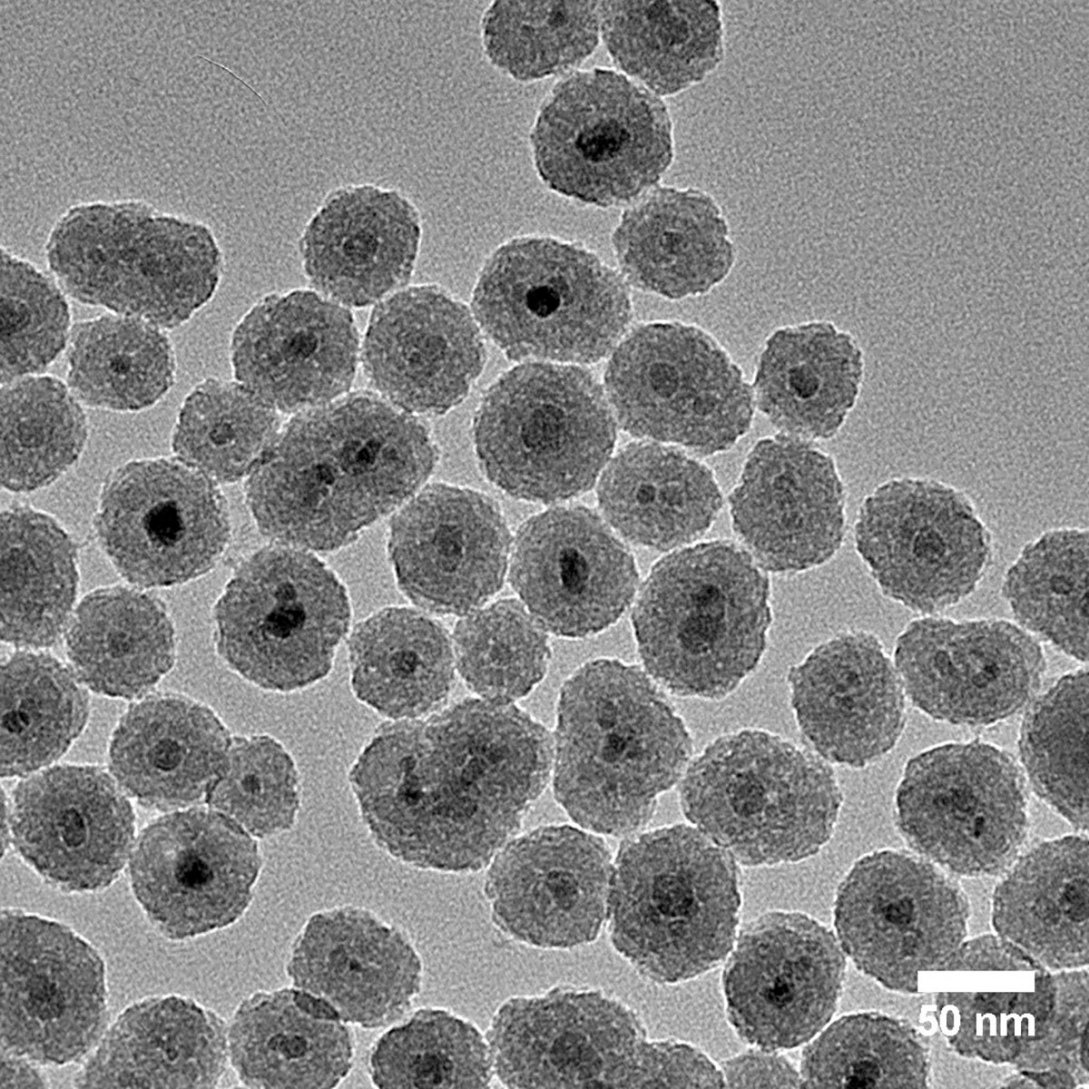
Representative TEM image of sIONP.

The detailed synthesis method of the sIONP is described by Gao et al. [12]. In short, 1.44g stock IONPs (EMG308, Ferrotec) was coated with 48 g polyvinyl pyrrolidone 10K (PVP, Sigma Aldrich) in 432 mL of MiliQ water by probe sonication. The silica shell was then coated on top of the PVP layer by Stöber method in the mixture of 3.2 L ethanol (Pharmco-Aaaper) and 160 mL ammonia (Avantor Performance Materials) [29]. Eighty milliliters of the silica shell precursor, tetraethylorthosilicate (TEOS, Sigma Aldrich) was added into the reaction while stirring at room temperature. After 1 h, 20 mL of 2-[methoxy(polyethyleneoxy)-propyl]9-12-trimethoxysilane (PEG-silane, Gelest) was added to the reaction. After an additional 0.5 h, 3 mL of Chlorotrimethylsilane (TMS, Sigma Aldrich) was added to the reaction. The reaction continued overnight and the resulting sIONP solution was purified by repeated centrifugation and washing with decreasing concentrations of ethanol in water, and eventually by dispersion in water. The sIONP solution was characterized by TEM, DLS, Zeta potential and ICP-OES for quality control, Fig 3.

## Results and discussion

**Fig 4:**
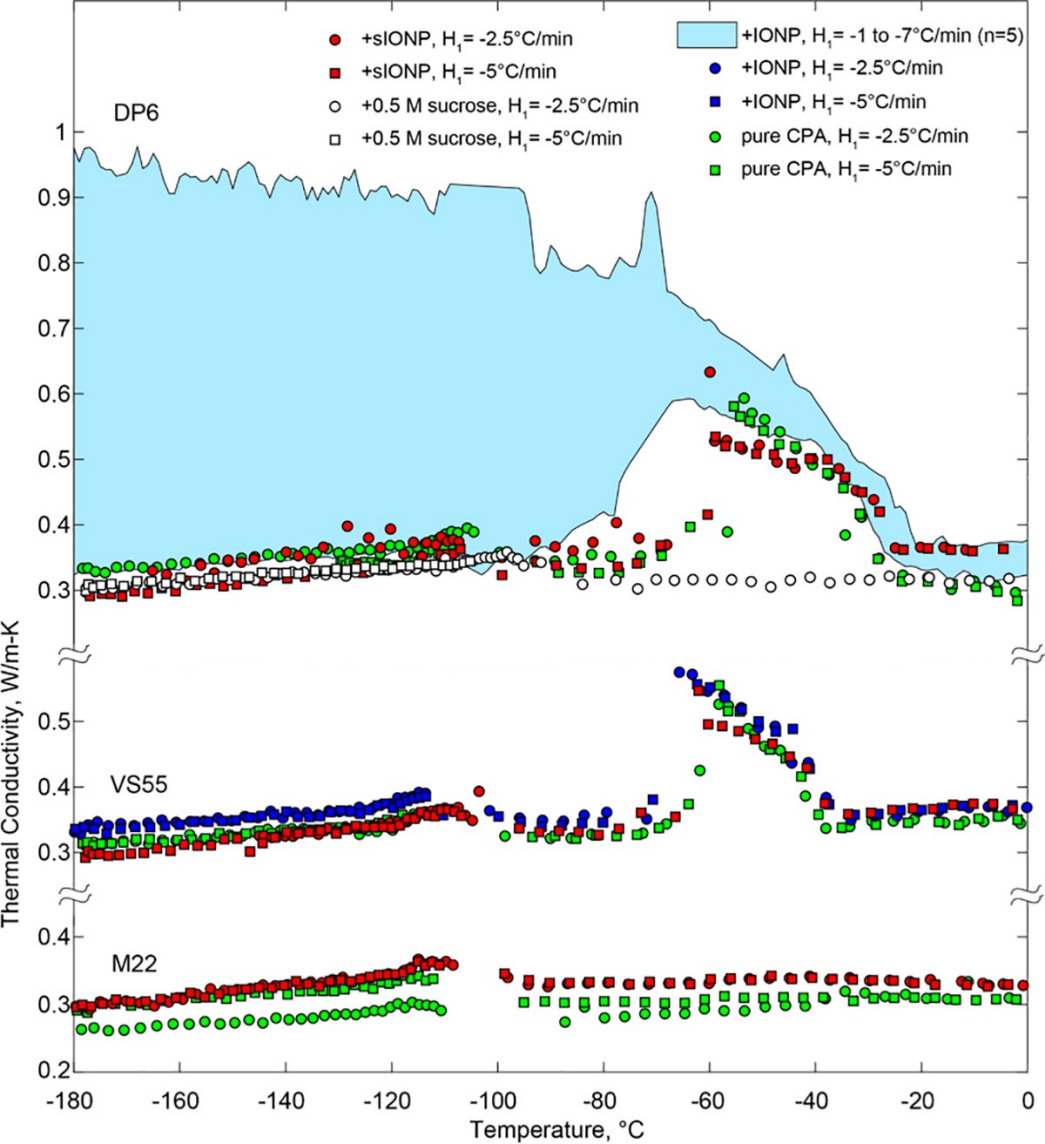
Thermal conductivity measurements of selected CPAs and nanofluids combinations; *H*_1_ refers to the initial cooling rate between 12ºC and -100ºC, where measurements were taken during passive warming to -100°C and at 3°C/min thereafter.

**Fig 5:**
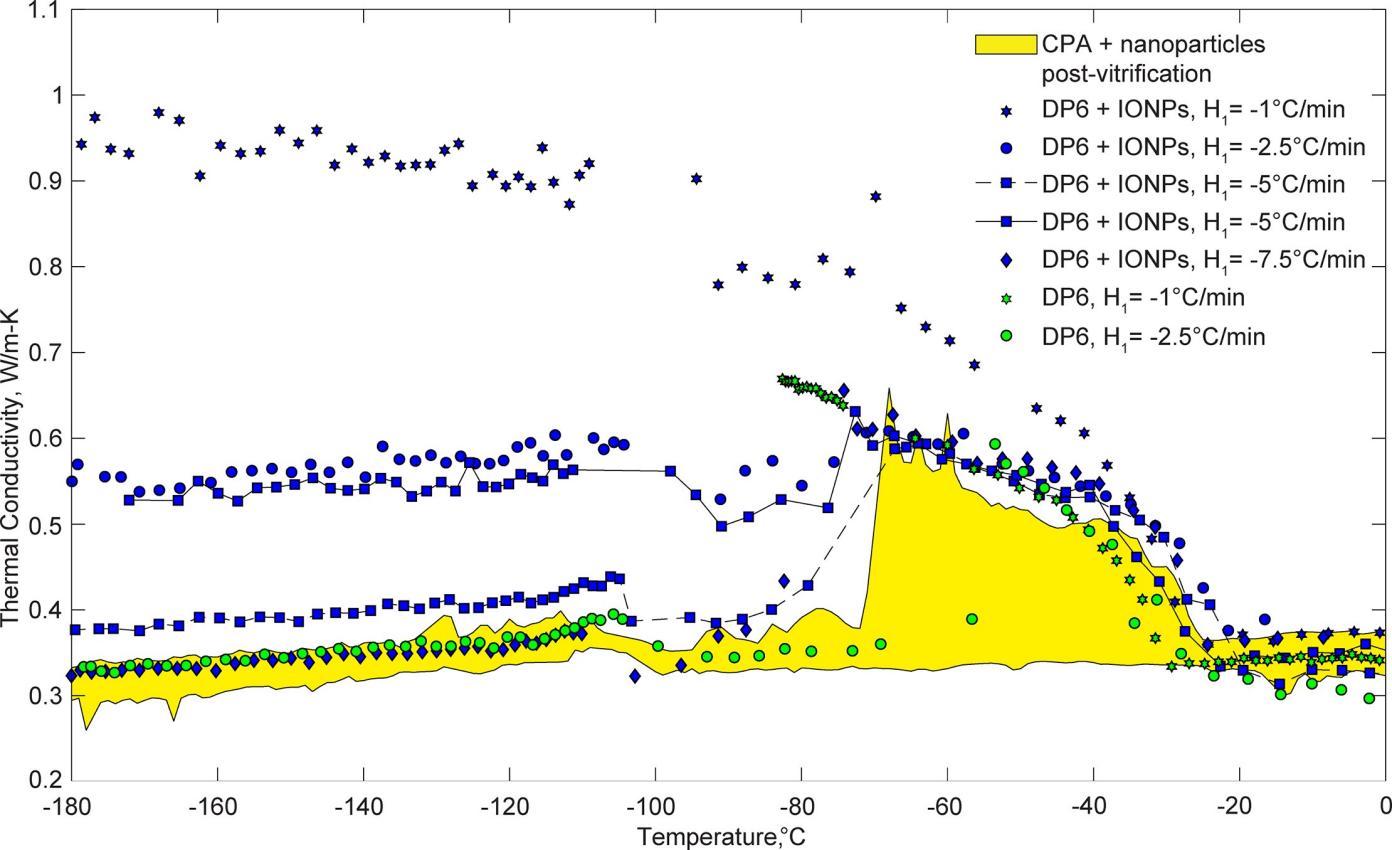
Selected thermal conductivity measurements for DP6+IONP, where all other CPAs and nanofluids combinations were found within the yellow range; *H*_1_ refers to the initial cooling rate between 12ºC and -100ºC, where measurements were taken during passive warming to -100°C and at 3°C/min thereafter. Pure DP6 was measured previously and is displayed here for reference [13, 18].

Fig 4 displays selected results for previously measured thermal conductivity of pure DP6 [14], DP6+sucrose, DP6+IONP, DP6+sIONP, pure VS55, VS55+IONP, VS55+sIONP, pure M22, and M22+sIONP. While DP6+IONP is displayed as a range in Fig 4, the results of individual experiments are displayed in Fig 5, which calls for a more detailed discussion being a primary target of investigation due to its potential for favorable cryopreservation conditions. Table 1 lists polynomial fit coefficients for all experimental data displayed in Figs 4 and 5. Those coefficients were determined by the Matlab polyfit function, using a built-in least squares method, where the order of the polynomial was selected by the authors. These polynomial approximations are provided so that they may be used in future studies focusing on cryopreservation simulations and analyses.

**Table 1:** Best-fit polynomial approximation coefficients for thermal conductivity data displayed in Figs 4 and 5, where average values are listed in cases where the span of the polynomial approximation is smaller than two standard deviations of the experimental data over the relevant temperature range (denoted by †).

CPA	Additive	H_1_	Temperature Range, °C	*a*_0_	*A*_*1*_	*a*_*2*_	*a*_3_	*a*_4_	R^2^
VS55	None	-2.5	-180 … -119	4.08×10^−1^	5.49×10^−4^				0.821
			-98 … -72 and -37 … 0	3.53×10^−1^	3.51×10^−4^				0.900
		-5	-180 … -125	4.03×10^−1^	5.05×10^−4^				0.920
			-93 … -73 and -38 … 0	3.52×10^−1^	3.27×10^−4^				0.936
	14 mg Fe/mℓ solution (IONP)	-2.5	-180 … -121	4.40×10^−1^	5.81×10^−4^				0.895
			-101 … -71	3.56×10^−1^**†**	-				-
			-32 … 0.0	3.71×10^−1^	5.22×10^−4^				0.794
		-5	-180 … -121	4.39×10^−1^	5.95×10^−4^				0.930
			-102 … -77 and -35 … 0	3.72×10^−1^	2.91×10^−4^				0.704
	11 mg Fe/mℓ solution (sIONP)	-2.5	-180 … -123	4.30×10^−1^	7.30×10^−4^				0.829
		-5	-180 … -116	4.40×10^−1^	8.16×10^−4^				0.927
			-96 … -77 and -34 … 0	3.74×10^−1^	4.59×10^−4^				0.892
M22	None	-2.5	-180 … -121	3.59×10^−1^	5.72×10^−4^				0.499
			-87 … 0	3.21×10^−1^	4.82×10^−4^				0.718
		-5	-180 … -116	3.99×10^−1^	5.82×10^−4^				0.938
			-95 … 0	3.11×10^−1^	8.48×10^−5^				0.415
	11 mg Fe/mℓ solution (sIONP)	-2.5	-180 … -119	4.45×10^−1^	8.31×10^−4^				0.965
			-91 … 0	3.34×10^−1^**†**	-				-
		-5	-180 … -121	4.55×10^−1^	9.08×10^−4^				0.781
			-105 … -61	3.33×10^−1^**†**	-				-
DP6	0.5 M Sucrose	-2.5	-180 … -108	3.91×10^−1^	4.96×10^−4^				0.887
			-78 … 0	3.14×10^−1^**†**	-				-
		-5	-180 … -114	3.97×10^−1^	5.12×10^−4^				0.939
	18 mg Fe/mℓ solution (IONP)	-1	-180 … -26	-7.89×10^−2^	-2.35×10^−2^	-2.20×10^−4^	-9.41×10^−7^	-1.49×10^−9^	0.956
			-25 … 0	3.75×10^−1^	5.49×10^−4^	-	-	-	0.414
		-2.5	-180 … -104	6.62×10^−1^	6.51×10^−4^	-	-	-	0.661
			-68 … -21	4.78×10^−2^	-2.42×10^−2^	-4.22×10^−4^	-3.44×10^−6^	-9.57×10^−9^	0.951
		-5	-180 … -118	4.70×10^−1^	5.12×10^−4^				0.735
			-17 … 0	3.24×10^−1^	-4.49×10^−4^				0.645
		-5	-180 … -116	5.44×10^−1^**†**	-				-
			-23 … 0	3.54×10^−1^	5.73×10^−4^				0.572
		-7.5	-180 … -112	4.24×10^−1^	5.50×10^−4^				0.936
			-24 … 0	3.64×10^−1^**†**	-				-
	11 mg Fe/mℓ solution (sIONP)	-2.5	-180 … -116	5.28×10^−1^	1.20×10^−3^				0.562
		-5	-180 … -112	4.29×10^−1^	7.82×10^−4^				0.943
			-92 … -73 and -27 … 0	3.64×10^−1^	3.23×10^−4^				0.833

The fit for thermal conductivity is in the form *k* = *a*_4_*T*^4^+*a*_3_*T*^3^*+a*_2_*T*^2^+*a*_1_*T*+ *a*_0_.

The data gap around -100°C for some individual runs (Figs 4 and 5) is associated with the transition from passive rewarming to an active rewarming control (Fig 2), resulting in a controlled variable overshoot for a short period [13, 14]. Since the certainty in thermal conductivity measurements during the overshoot period is unknown, the corresponding data segment is omitted from presentation.

### Cryomacroscopy of nanofluids

Consistent with the literature of recent years, a water-based solution containing nanoparticles and the term *nanofluid* are used interchangeably in this report. Pure CPAs are translucent in their liquid and vitrified states (glass is essentially a liquid in an arrested state due to extreme viscosity), allowing physical effects such as crystallization and fracture formation to be observed during the cryoprotocol [13, 14]. However, due to their optical properties, nanoparticles in the concentrations used in the current study turn the CPA cocktail (now, nanofluid) opaque and brownish. For that reason, using cryomacroscopy to study physical effects in the CPA+nanoparticles system has its limitations. Here, only surface effects can be observed, which could serve as indication of volumetric and/or internal events. For example, Fig 2(C) displays crystallization in pure VS55, while Fig 2(B) displays fractures at the surface of the sample in the case of DP6+IONP. The fractures in the latter case were generated when the sample was contained in a quartz cuvette (Thorlabs, 3500 μℓ Macro Quartz Cuvette), but could be avoided when using a plastic PMMA cuvette, with the latter having thermal expansion coefficient similar to the vitrified CPA cocktail [30]. As discussed before [14] and addressed below, crystallization events can alternatively be identified solely from thermal conductivity measurements.

### Vitrification, rewarming-phase crystallization, and thermal conductivity

Of the CPA cocktails tested in this study, M22 has the highest solutes concentration and, hence, the lowest critical cooling rate (CCR), which is a cooling-rate threshold above which vitrification is achieved. Based on a recent differential scanning calorimetry (DSC) study, the CCR of M22 is not higher than 0.1°C/min [31]. No crystallization events have been observed with M22 in the current study, where the thermal conductivity of the amorphous state can be several folds lower than the thermal conductivity of the higher-order, crystalline material. Ehrlich et. al [32] has demonstrated the effects of such thermal conductivity variations on thermal analyses of kidney cryopreservation. Fig 4 displays the low variation in thermal conductivity in the temperature range of measurement (-180°C to 0°C) across repeated measurements of M22. It can also be observed there that M22 has the lowest thermal conductivity when compared with the other CPA cocktails tested, whether loaded with nanoparticles or not.

VS55 has the second highest concentration of solutes, having CCR of -2.5°C/min [33], but unlike M22, it does not contain synthetic ice blockers. Results displayed in Fig 4 suggest that the thermal conductivity of VS55 and M22 is in a close range in the vitrified state. The increased thermal conductivity of VS55 between -70°C and -40°C is indicative of rewarming-phase crystallization (RPC). The term RPC is used in this report as an all-inclusive term addressing both devitrification (rewarming-phase ice nucleation and growth) and ice growth from ice crystals already formed during cooling. Recall that thermal conductivity in this study is measured during the rewarming phase of the cryogenic protocol. The observation that results from all experimental conditions tested with VS55 display similar thermal conductivity values at -180°C suggests that the corresponding protocols led to vitrification. While ice nucleation near the glass transition temperature during cooling cannot be ruled out, the behavior of the thermal conductivity during rewarming suggests that RPC in this case is essentially associated with devitrification. Either way, eliminating the RPC is a major motivation behind the integration of nanoparticles with the CPA cocktail. Unfortunately, RF heating could not be applied in this study, which would likely prevent RPC but would obstruct the hot-wire measurements.

Based on cryomacroscopy observations, some ice crystals formed in VS55 experiments on the solution surface and at the interface with the cuvette cap. These were observed after a cooling rate of -2.5°C/min and -5°C/min. Despite those visual observations the thermal conductivity remained unaffected, which could be explained by the fact that the crystals were observed on the outer surface of domain only while the hot-wire sensor measures at the center of the domain, where complete vitrification was evident. This represents inconsistency between visual evidence of crystallization at -5°C/min and the previously reported VS55 CCR of -2.5°C/min in the microliter-size sample in DSC studies. It is feasible that the increase in sample size increases the likelihood of crystallization [34–36], which is consistent with previous studies [20].

DP6 has the lowest solutes concentration tested in this study, which is associated with a lower toxicity potential. For that reason, it received the highest attention in terms of range of conditions and nanoparticles combinations. This research emphasis is consistent with an ongoing study to combine DP6 with a battery of synthetic ice modulators (SIMs) [22, 23, 26, 37], in order to improve its glass forming tendency while maintaining a low toxicity potential. Of all the DP6 and SIMs combinations tested previously, DP6+sucrose has been demonstrated most favorable to cryopreservation of blood vessels [38, 39], in conditions which are relevant to large-size tissues and organs. Interestingly, glucose has also been found to dramatically affect the vitrification tendency of DP6, in a study focusing on the contribution of vehicle solutions to physical effects occurring during vitrification [14]. With those observations in mind, the study on vitrification tendency and stability of DP6 for complex-tissue cryopreservation has also been recently presented [28]. Note that the SIBs in M22 could be considered as a subset of the broader category of SIMs [23].

It can be seen from Fig 4 that DP6+0.5M sucrose does not display RPC, and also that the thermal conductivity values are in close range with that of M22. Note that the CCR of DP6+0.5M sucrose is less than 1°C/min [28]. Further note that the overall concentration of glass promoting agents in DP6 and VS55 is 6M and 8.4M, respectively (40% difference!). Comparing the thermal conductivity behavior of VS55 and DP6 demonstrates the dramatic effect of low sucrose concentration on the cocktail. A relatively wider range of DP6 experiments were conducted in this study since it is considered a promising base solution for the inclusion of SIMs, where additional results are displayed in Fig 5.

### The effects of IONP vs sIONP on the CPA cocktail

The addition of sIONP to any of the CPA cocktails tested in this study did not significantly affect the thermal conductivity, its tendency to vitrify or, conversely, to form RPC, as can be observed from Fig 4. Note that only sIONP are considered biocompatible for the purpose of cryopreservation, while IONP may adversely interact with the tissue.

While neither sIONP nor IONP appear to affect the thermal conductivity of VS55 compared with the pure solution, the thermal conductivity of DP6 is affected by the presence of IONP but not by the presence of the sIONP. For this reason, the range of thermal conductivity for the more crystallization-sensitive DP6+IONP is highlighted cyan in Fig 4, while a selection of individual runs is displayed in Fig 5. It can be observed from Fig 5 that the presence of IONP reduces the tendency to vitrify, where a sample that was cooled at -5°C/min displays an increased thermal conductivity at -180°C, well below the glass transition temperature (-115°C). Recall that the critical cooling rate of DP6 is -2.7°C/min [28]. Furthermore, repeated experiments at -5°C/min display variation in results up to twofold, indicating unstable conditions (highlighted with solid and dashed lines in Fig 5). Note that a cooling rate of *H*_1_ = -7.5°C/min resulted in thermal conductivity similar to that of the vitrified pure DP6 at low temperatures, which was also found within the range of all other solution types tested in this study in the vitrified state.

It is emphasized that the critical cooling rate reported in the literature is typically of pure microliter-size samples, while the current study examines macro-size samples. Such large samples statistically increase the potential for impurities, interface effects, and even mechanical stresses, all of which effectively elevate the cooling rate needed to prevent crystallization. This could explain the variability in thermal conductivity between similar experiments under the same conditions, subject to marginal conditions to suppress crystallization, such as with the DP6+IONP at -5°C/min (Fig 5). In a broader perspective, this observation supports the approach that experimental investigations on large samples is required when large-size cryopreservation is considered. This is in contrast of extrapolation from micro samples to large-size cryopreservation applications, whether relating to physical effects, such as crystallization and thermal conductivity, or when relating to biological effects.

### Effective thermal conductivity in partial crystallization

The three-fold thermal conductivity increase in Fig 5 for DP6+IONP is explained by the increasing amount of ice crystallization as the cooling rate decreases. Since a phase diagram is not yet available for DP6, one may estimate the ice crystal concentration of the CPA using the effective medium theory (EMT) model [40], which offers an estimation for the effective thermal conductivity of a randomly distributed two-component mixture:

$$\nu_{1}\frac{k_{1}-k_{e}}{k_{1}+2k_{e}}+\left(1-\nu_{1}\right)\frac{k_{2}-k_{e}}{k_{2}+2k_{e}}=0$$ (3)

where *k* is the thermal conductivity, *ν* is volume fraction, and the subscripts 1, 2, and *e* refer to the first component, the second component, and the effective property of the mixture, respectively.

In the current analysis, the vitrified DP6+IONP is taken as the first component, having the measured thermal conductivity of vitrified DP6+IONP (*H*_1_ = -7.5°C/min), while hexagonal ice crystals are taken for the second component, corresponding to a thermal conductivity of 3.8 W/m-K at -115°C [41], which is the glass transition temperature. Finally, the value of ν_1_ is estimated such that the predicted value *k*_e_ matches the measured thermal conductivity of the partially crystallized DP6+IONP. For example, the amount of crystals estimated for the slowest cooling rate of *H*_1_ = -1°C/min is 33% (i.e., ν_1_ equals 0.67) at -115°C. This is a significant observation where only 33% of the medium is ice, but it increases the thermal conductivity by three-fold. Note that the thermal conductivity of ice crystals increases with the decreasing temperature in the relevant temperature range, while the thermal conductivity of the vitrified material displays an opposing trend of decreased value with the decreasing temperature within the same range. This explains the trend of the effective thermal conductivity below the glass transition temperature, when no additional crystallization is expected.

VS55+IONP exhibited a slightly increased thermal conductivity as compared with pure VS55. For example, at -115°C the thermal conductivity of VS55+IONP is 8% higher than that of pure VS55. Since this trend was not sensitive to cooling rate, as was evident with DP6+IONP in comparison with pure DP6, it is concluded that the corresponding thermal conductivity difference is not related to crystallization but to the presence of nanoparticles.

### Effective thermal conductivity of the nanofluid

DP6+IONP and VS55+IONP nanofluids were synthesized with 18 mg Fe/mℓ solution and 14 mg Fe/mℓ solution, respectively (0.70% and 0.54% iron oxide by volume). Here, the Maxwell-Eucken (ME) model [40] is selected to predict effective thermal conductivity of CPA+IONP, where the physical structure assumed is that of a continuous medium containing small spheres evenly distributed throughout. The spheres are assumed to be sufficiently small and far apart from one another such that local thermal fluctuations surrounding each sphere do not affect temperature of neighboring spheres [40]. This model does not consider any phase change, or possible settling or aggregation of nanoparticles after sonication. This model also does not account for the thermal boundary resistance between the CPA and the nanoparticles.

The ME model was chosen to approximate the effective thermal conductivity of the vitrified nanoparticle solutions rather than the EMT model because of the topology features of the sonicated nanoparticle solutions, which are not a randomly distributed two-component medium. By contrast, the EMT model was chosen to approximate the effective thermal conductivity of the partially crystalline DP6+IONP solution because (i) ice crystals may or may not grow in completely random orientations and clusters, and (ii) the ME model is not valid for high sphere volume fraction (ν_sphere_ <25%) [42].

According to the Maxwell-Eucken model, the effective thermal conductivity is expressed as:

$$k_{e}=\frac{k_{1}v_{1}+k_{2}v_{2}\frac{3k_{1}}{2k_{1}+k_{2}}}{v_{1}+v_{2}\frac{3k_{1}}{2k_{1+}k_{2}}}$$ (4)

Here, the first component is the continuous phase of pure CPA, while the second component is the iron oxide particles.

The thermal conductivity of the pure CPA component is taken a temperature of -125°C and cooling rate of *H*_1_ = -2.5°C/min (see also [14]). The thermal conductivity of the iron oxide component is taken as 5.5 W/m-K, which is approximated as the average between thermal conductivities of magnetite (Fe_3_O_4_) and hematite (Fe_2_O_3_) at 0°C [43]. Although the Fe_3_O_4_ and the Fe_2_O_3_ have different thermal conductivities, the effective thermal conductivity of CPA+IONP, *k*_e_, was found to be insensitive to the value of thermal conductivity of the nanoparticles due to their low volume fraction. For the CPA+sIONP, the effective property of the spheres was taken as that of the silicon dioxide coating, where the nanoparticle core is much smaller than the coating volume (see Fig 5 for example and recall that the volume ratio is the diameter cube ratio). The volume ratio of the silicon dioxide is about 7%, and a thermal conductivity of 0.8 W/m-K was assumed at -125°C [44]. Here, the iron oxide core volume ratio is only 0.21% and is therefore omitted from calculations.

**Fig 6:**
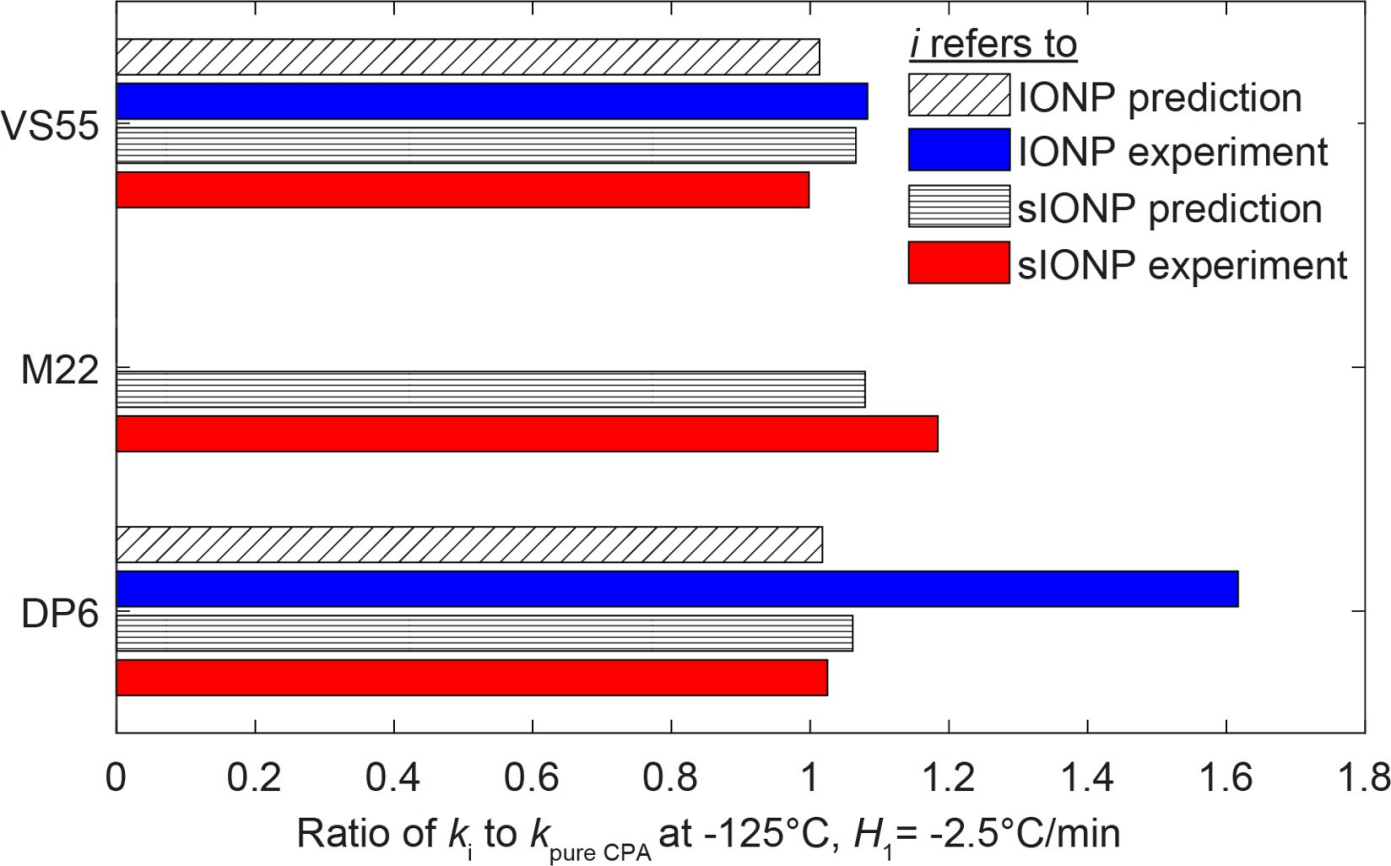
Ratio of thermal conductivity of nanofluids to thermal conductivity of the pure CPA at -125°C (below glass transition temperature) for a cooling rate of H1 = -2.5°C/min; predictions are based on the Maxwell-Eucken model.

Fig 6 displays the ratio of thermal conductivity of the nanofluid to the thermal conductivity of the pure CPA for various solution types, based on the ME model. As could be expected, the thermal conductivity of nanofluids is always greater for nanofluids than the respective pure CPA. With the exception of DP6+IONP, the thermal conductivity difference between predicted values and measured data is within 10%. This difference exceeds 60% for the case of DP6+IONP, which suggests that other effects are involved, with ice crystallization as the obvious suspect. This observation suggests that IONP may promote crystallization.

## Summary and conclusions

Thermal Conductivity of various CPA solutions have been measured in the presence and absence of the nanoparticles IONP and sIONP, using the transient hot-wire technique. All pure CPA cocktails tested vitrified under the applied cooling rates. No crystallization events were observed during rewarming of M22 or DP6+0.5M sucrose from cryogenic storage temperatures, while VS55 displayed some rewarming-phase crystallization RPC, which can be identified solely from thermal conductivity measurements.

The addition of sIONP to any of the CPA cocktails tested in this study did not significantly affect the thermal conductivity, its tendency to vitrify or, conversely, to form RPC. Fractures were observed at the onset of rewarming for DP6+sIONP using cryomacroscopy, despite the inherent opacity of the sIONP solutions. The fractures occurred under carefully planned conditions, where the cooling rate was relatively high (-20°C/min) to the solutions used, while holding the samples in a quartz cuvette rather than the standard PMMA cuvette. It is likely that using RF heating in order to accelerate the rewarming rate and to unify the temperature distribution would prevent fracture and RPC. However, sIONP were not activated in this study, as the RF heating mechanism would interfere with thermal conductivity measurements.

The addition of IONP to DP6 affected crystallization, where the outcome was sensitive to cooling rates in the range of -2.5°C/min to -5°C/min. A cooling rate of -1°C/min yielded thermal conductivity of 0.95 W/m-K at low temperature, a three-fold increase from that of pure vitrified DP6 at low temperature. The addition of IONP to DP6 appears to hinder the tendency of the CPA to vitrify, which is a detrimental effect. But it is unlikely that uncoated nanoparticle solutions will be used in practical applications. This is because uncoated nanoparticles are less stable in the solution and may aggregate or settle in CPA [9]. In general, the addition of sIONP to CPA did not affect thermal conductivity, nor did it affect RPC, which are encouraging observation in the development of nanofluids-based CPA cocktails for ice-free cryopreservation.
